# Dyspepsia and the Teeth

**Published:** 1880-04

**Authors:** 


### DYSPEPSIA AND THE TEETH.

I have met dental practitioners many times who seemed to be unaware that the teeth bear any close relation to the stomach. Some I have met, who laughed at the idea so often found in the minds of patients, that certain teeth are related to the stomach; by the patient these are called stomach teeth. The fact is that all the teeth are stomach teeth, placed at the entrance of the alimentary canal to prepare food for gastric digestion and possessing very extraordinary sympathies with not alone the stomach, but the whole alimentary canal.

The condition of the teeth must exert a favorable or unfavorable influence on the stomach; so also the condition of the stomach must exert a favorable or unfavorable influence on the teeth. I am astonished to find myself formally stating a matter which is so abundantly recognized by all who have made observations in this direction. It has however fallen to my lot to meet with many practitioners, who not only seem blind to this fact, but whose patients are constantly discharged without having received appropriate treatment.

The man who fills a simple cavity and sends away his patient with an acid saliva, corroding and dissolving all the organs of mastication from week to week or from month to month, may believe that he has done his duty, but he has not. He has left the most important thing undone; he should have rendered that saliva, normally alkaline, by appropriate treatment. Does he say that it is none of his business to treat the constitutional condition of his patients, and that this is the work of the physician? I say it is not the work of the physician. In this instance it is the work of the dentist, and that the man who habitually leaves this work undone is not to be trusted as a dental practitioner. He is not a doctor—not a pathologist. He is a mere mechanic; he is a handicraft-man, a tooth carpenter, a quack.

I should have supposed that every practitioner of dentistry must be familiar with the fact that the saliva is normally alkaline. I should have always believed this with never a doubt to disturb my confidence, had I not found a number of country dentists and several city practitioners who had no idea as to whether the saliva is either normally acid or alkaline, or both, or neither. Strongly enough I have seen the doctrine advocated that the saliva is normally acid, whether this investigator regarded a condition of acidity as not unfavorable to the teeth, (a most false view of the case,) or whether he regarded it as a beautiful provision of divine Providence, whereby, dentists might have cavities to fill in the teeth of the community, is not on record, and I have no means of knowing. It is enough for us to understand once for all, that the normal saliva is alkaline, and that when it is so, it tends to the preservation of the teeth which it bathes.

But the saliva is not always normal, with test paper at hand, any practitioner may satisfy himself that in more than half of his patients the saliva is neutral or acid, it is almost invariably acid in dyspeptics, *sour* is the word for both saliva and stomach, and what shall we say of the man who abuses the confidence of his patient, by merely repairing such lesions as an abnormal saliva have produced or permitted, without warning him that he must change his habits or modes of life, or take some treatment whereby he shall free his teeth from the action of an ever present, ever corroding fluid, the practitioner who is derelict in his duty is a guilty man, if he is ignorant of his fault, I hope that God will forgive him, if he is not ignorant of his flagrant breach of a confidence which his patient has reposed in him, I hope also that God will forgive him, in this world he will find few who can excuse either the educated delinquent, or the ignorant pretender.

For my own part, I never want to meet another dentist who does not at least *desire* to so put the general system of his patient to right, that its constitutional vices and disorders shall either cease to exist or shall at least cease to act unfavorably on the teeth.

